# Supplementary material for: The future landscape of large language models in medicine
Source: Commun Med (Lond). 2023 Oct 10;3:141. doi: 10.1038/s43856-023-00370-1 (PMC10564921; doi:10.1038/s43856-023-00370-1)
Supplement: Supplementary file 1 — Description of Additional Supplementary Files [file 43856_2023_370_MOESM1_ESM.pdf]

Description of Additional Supplementary Files

File Name: Supplementary Data

Description: Example Prompts and outputs of GPT-3.5 and GPT-4 in the context of patient care, research, and medical education. Model outputs were generated using ChatGPT (model versions: GPT-4 – May 3, 2023, GPT-3.5 – May 3, 2023).
